# The Early Childhood Development of Pediatric Burn Patients

**DOI:** 10.3390/ebj5020012

**Published:** 2024-05-14

**Authors:** Maxime D. Cuijpers, Moniek Akkerman, Martin G. A. Baartmans, Paul P. M. van Zuijlen, Anouk Pijpe

**Affiliations:** 1 Burn Center, Red Cross Hospital, Vondellaan 13, 1942 LE Beverwijk, The Netherlands; ppmvanzuijlen@me.com; 2 Department of Plastic, Reconstructive and Hand Surgery, Amsterdam UMC—VU University Amsterdam, Boelelaan 1117, 1081 HV Amsterdam, The Netherlands; 3 Tissue Function and Regeneration, Amsterdam Movement Sciences, Boelelaan 1117, 1081 HV Amsterdam, The Netherlands; 4 Association of Dutch Burn Centers, Zeestraat 27-29, 1941 AJ Beverwijk, The Netherlands; m.akkerman@mzh.nl; 5 Burn Center, Martini Hospital, Van Swietenplein 1, 9728 NT Groningen, The Netherlands; 6 Research Group for Healthy Ageing, Allied Health Care and Nursing, Hanze University of Applied Sciences, Eyssoniusplein 18, 9714 CE Groningen, The Netherlands; 7 Department of Pediatrics, Maasstad Hospital, Maasstadweg 21, 3079 DZ Rotterdam, The Netherlands; baartmansm@maasstadziekenhuis.nl; 8 Burn Center, Maasstad Hospital, Maasstadweg 21, 3079 DZ Rotterdam, The Netherlands; 9 Department of Plastic, Reconstructive and Hand Surgery, Red Cross Hospital, Vondellaan 13, 1942 LE Beverwijk, The Netherlands; 10 Department of Pediatric Surgery, Emma Children’s Hospital, Amsterdam UMC—University of Amsterdam, Meibergdreef 9, 1105 AZ Amsterdam, The Netherlands

**Keywords:** burns, children, development

## Abstract

Our study aimed to provide a description of the early childhood development of pediatric burn patients relative to Dutch reference values, using both pre- and post-burn data from the Dutch Development Instrument and the *D*-score. Data from the Dutch Development Instrument were used to calculate the *D*-score and age-standardized *D*-score. Similar to a growth chart, the *D*-score was used to plot pediatric burn patients’ development relative to Dutch reference values for their age. Pediatric burn patients’ (n = 38) median age at the time of injury was 1.0 (1.0–2.0) years old. Burn size ranged from 1.0% to 36.0% of the total body surface area. Ninety-five percent (± 6.0%) of pediatric burn patients passed each of the age-appropriate developmental milestones at the target age. The mean age-standardized *D*-score was just above the Dutch average (+0.49 SD [0.18, 0.80]) and did not vary depending on sex (*p* = 0.06) or burn size (*p* = 0.41). In conclusion, among pediatric patients aged up to two-and-a-half years old, with non-full thickness burns, development was on track relative to the Dutch reference values. Our findings offer valuable first insights into the early childhood development of pediatric burn patients and may alleviate some parental concerns.

## 1. Introduction

Burns are among the top five most common causes of non-fatal childhood injuries worldwide [1]. In the Netherlands alone, almost three hundred children are admitted to one of three specialized burn centers every year [2,3]. Early childhood, from birth until four years old, is associated with an increased risk of burns [2,4]. Early childhood is characterized by the development of perceptual, motor, cognitive, language, socio-emotional, and self-regulation abilities that form the foundation for future health, well-being, education, and productivity [5]. During this time, children’s newfound and independent mobility allows them to explore their surroundings. However, children’s curious nature exceeds their cognitive ability to detect and avoid safety hazards [6,7,8]. Meanwhile, parents and caregivers often underestimate children’s capabilities and extent of reach [9].

On the other hand, during early childhood, children’s development is most susceptible to environmental influences, including health, nutrition, safety, caregiving, and early learning opportunities [5,10,11]. For example, children who have been diagnosed with a chronic physical illness or cancer have an increased risk of impaired physical, social, emotional, and cognitive functioning [12,13,14]. These functional impairments are not just a result of the condition itself, but also of the stressful events (e.g., hospitalization) and environmental changes (e.g., reduced opportunity for social interaction with peers) associated with the condition [15].

A review of the empirical literature by Bakker et al. (2013) identified two studies that addressed the post-burn development of pediatric burn patients [16,17,18]. First, Gorga et al. (1999) reported on the physical, functional, and developmental outcomes of pediatric burn patients aged between six months and six years old, with a burn covering, on average, 6.0% (± 4.5) of the total body surface area [17]. Pediatric burn patients’ developmental status was assessed using the Denver Home Screening Questionnaire, Denver Developmental Screening Test, and the Gross Motor Index and Fine Motor Index of the Peabody Developmental Motor Scale [19,20,21]. All outcome measures were assessed at one-month, six-months, and one-year post-burn. Gorga et al. reported an increase in the number of pediatric burn patients with a suspected developmental delay, in particular within the domain of language. Beyond the impact of the burn-related hospitalization, however, this increase could be attributed to the presence of both an increased risk of developmental delay and pre-existing developmental delay within the cohort.

Second, Nayeb-Hashemi et al. (2009) reported on the cognitive and affective outcomes of pediatric burn patients aged up to eighteen years old, with a full-thickness burn of the skull [18]. Cognitive and affective status was assessed using clinical information from interviews and observations, parental reports, and psychological and psychiatric evaluations, available from the pediatric burn patients’ medical records. Nayeb-Hashemi et al. reported acute developmental delay within the domains related to motor function, communication, and vision among pediatric burn patients aged up to three and a half years old.

More recently, Kazis et al. (2016) reported on the post-burn recovery and development of pediatric burn patients aged up to five years old, with a burn covering, on average, 18.0% (±18.1) of the total body surface area [22]. Post-burn recovery and development was assessed using the Burn Outcomes Questionnaire (BOQ_0–5_) between one-month and four-years post-burn [23]. The BOQ_0–5_ is a questionnaire developed to monitor the health-related quality of life of pediatric burn patients based on parent-reported outcomes of physical, emotional, and social recovery [22,23]. Kazis et al. reported that pediatric burn patients surpassed the norm within the BOQ_0–5_ domains related to language, emotional behavior, and family functioning. Nevertheless, significant deficits were observed within the BOQ_0–5_ domains related to play, fine motor skills, gross motor skills, pain or itching, appearance, satisfaction with care, and worry or concern.

The body of evidence on the post-burn development of pediatric burn patients is characterized by a limited number of studies. Furthermore, the studies demonstrate methodological shortcomings, including a lack of information on the pre-burn developmental status and the use of a health-related quality of life instrument. Our study aimed to provide a description of the early childhood development of pediatric burn patients relative to Dutch reference values, using both pre- and post-burn data from the Dutch Development Instrument and the *D*-score, a single measure that quantifies the development at a given age and allows for cross-cultural comparison.

## 2. Materials and Methods

### 2.1. Study Population

This study is part of a larger retrospective cohort on pediatric patients’ post-burn growth. Children of all ages who either sustained a burn requiring hospital admission of more than one day or surgical treatment at one of the Dutch burn centers in 2013, or a severe burn in 2009–2018, were initially enrolled (n = 112) [24]. A severe burn was defined as a burn covering more than ten percent of the total body surface area. Exclusion criteria included: (i) death during or after the hospital admission; (ii) incomplete or outdated contact information; (iii) living outside of the Netherlands; and (iv) insufficient command of the Dutch language. Early childhood development was investigated in a subgroup of this cohort, consisting of children aged up to two-and-a-half years old at the time of injury (n = 59).

Written informed consent was provided by the legal guardian(s) or the patient. The medical ethics review committee of VU University Medical Centre ruled that the Medical Research Involving Human Subjects Act (WMO) was not applicable. The medical ethics review committee of VU University Medical Centre is registered with the US Office for Human Research Protections as IRB00002991 (Federal Wide Assurance: FWA00017598). This study was further approved by the institutional review boards of the participating hospitals: the Red Cross Hospital in Beverwijk, the Maasstad Hospital in Rotterdam, and the Martini Hospital in Groningen.

### 2.2. Data Collection: Demographic and Clinical Characteristics

Pediatric burn patients’ demographics and clinical characteristics were derived from the Dutch Burn Repository (NBR-R3), including age at the time of injury, sex, burn size, extent of full-thickness burn, etiology, date of injury, date of admission, length of hospital stay, number of surgeries, nutrition support, mechanical ventilation, and reconstructive surgery [3].

### 2.3. Data Collection: Dutch Development Instrument

Developmental data were collected from patients’ Child and Youth Health Care records (Dutch: Jeugdgezondheidszorg). Child and Youth Health Care is a free-of-charge nationwide program that aims to monitor children’s health and development. Children are invited for eighteen regular check-ups from birth until eighteen years old and attendance is voluntary. Nevertheless, the average reach of Child and Youth Health Care is 95.0% among Dutch children aged up to four years old [25]. Record linkage was performed using the pediatric burn patient’s full name, date of birth, postal code of residence, and sex. From the Child and Youth Health Care records, we collected both pre- and post-burn data on the gestational age, medical history, and the Dutch Development Instrument. Based on the age distribution of our study population and the national legal time limit for storage of this type of health data, a priori, we expected that some of the records could not be retrieved. However, to maximize the amount of data for our analyses, this was not a study exclusion criterion.

In the Netherlands, the Dutch Development Instrument is routinely used to monitor the early childhood development of children aged up to two-and-a-half years old. The Dutch Development Instrument is a performance-based instrument consisting of seventy-five developmental milestones across three domains: (i) fine motor skills, adaptation, personality and social behavior; (ii) communication; and (iii) gross motor skills (Supplement S1). During each visit, a subset of age-appropriate developmental milestones is administered by a physician or trained nurse. On each developmental milestone within the subset, the child is assigned a score: pass, fail, or not administered. An age-appropriate subset of developmental milestones is constructed based on the likelihood that approx. 90% of children at the target age will pass each of the developmental milestones. If a child does not pass a developmental milestone, it is re-administered during the next visit.

### 2.4. Statistical Analyses

Baseline characteristics were described using summary statistics. Data from the Dutch Development Instrument were used to calculate the (i) average percentage of pediatric burn patients who passed each of the age-appropriate developmental milestones at the target age; (ii) the average percentage of developmental milestones passed per domain, hereafter referred to as the ‘success rate’; (iii) the *D*-score; and (iv) the age-standardized *D*-score.

The *D*-score is a single measure that quantifies development at a given age [26,27]. In addition, the *D*-score is compatible with a multitude of international instruments for child development and was adopted by the Global Scale for Early Development from the World Health Organization [28,29].

In order to calculate the *D*-score and age-standardized *D*-score, data from the Dutch Development Instrument were first entered in accordance with the Global Scale for Early Development’s nine-position schema [30]. Resulting in milestones that consisted of nine characters and reflected the following: (i) the instrument: Dutch Development Instrument (“ddi”); (ii) the developmental domain: fine motor skills, adaptation, personality, and social behavior (“fm”); communication (“cm”); or gross motor skills (“gm”); (iii) an administration code; and (iv) an item number.

Subsequently, a *D*-score and age-standardized *D*-score were calculated for each visit using the “dscore” package in R [26,27,31,32,33]. Calculation of the *D*-score was based on the pass/fail score on each of the developmental milestones that was administered, a difficulty estimate for each of the developmental milestones that was administered, and a prior distribution [26,27].

Similar to a growth chart, the *D*-score was then used to plot pediatric burn patients’ individual developmental curves relative to Dutch reference values from the Social Medical Survey of Children attending Child Health Clinics (SMOCC) [34]. Stratification further allowed us to explore differences in pediatric burn patients’ mean age-standardized *D*-score based on sex or burn size. Statistical analyses were performed using R version 4.2.1 [31].

## 3. Results

### 3.1. Study Population

Among the 112 pediatric burn patients who were initially enrolled in the cohort, there were 59 patients aged up to two-and-a-half years old at the time of injury. Child and Youth Health Care records were available for 38 out of 59 pediatric burn patients. Among these 38 pediatric burn patients, there were 25 (66.0%) male and 13 (34.0%) female (Table 1). The median (IQR) age at the time of injury was 1.0 (1.0–2.0) years old. Burn size ranged from 1.0% to 36.0% of the total body surface area, with a median (IQR) burn size of 6.0% (4.5–11.9). Pediatric patients with burns covering more than ten percent of the total body surface area accounted for 47.4% (n = 18) of the study population. Scald was the predominant cause of injury (n = 34; 89.5%). One patient was previously diagnosed with developmental delay (n = 1; 2.6%).

### 3.2. Dutch Development Instrument

On average, pediatric burn patients attended 8.2 (±1.9) check-ups at a Child and Youth Health Care location from birth until two-and-a -half years old. Ninety-five percent (±6.0%) of pediatric burn patients passed each of the age-appropriate developmental milestones at the target age. Per domain of the Dutch Development Instrument, pediatric burn patients’ success rate was 88.0% (±19.0%), 81.0% (±19.0%), and 78.0% (±21.0%) on the age-appropriate developmental milestones related to (i) gross motor skills, (ii) communication, and (iii) fine motor skills, adaption, personality, and social behavior, respectively.

### 3.3. D-Score

**Figure 1:**
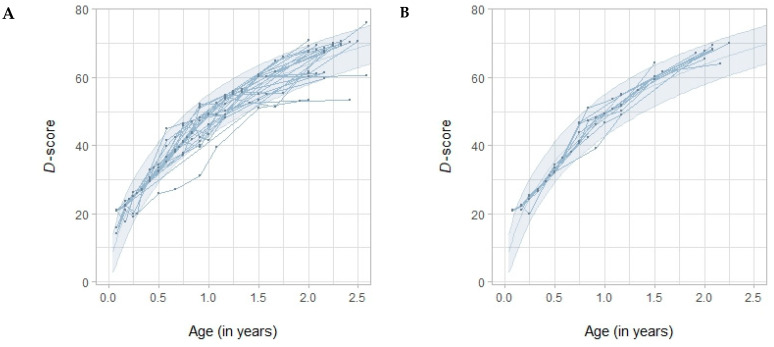

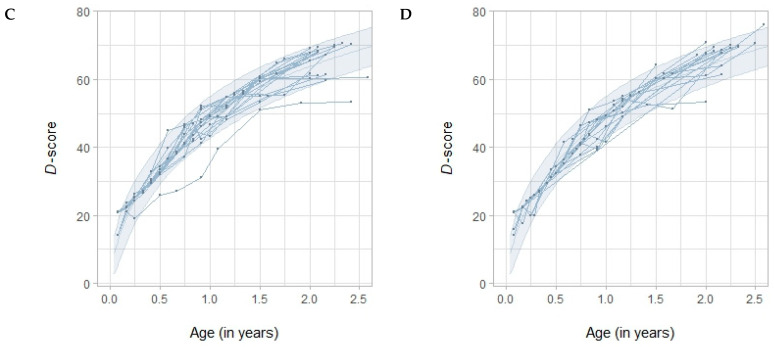
Developmental curves relative to the Dutch reference values of pediatric burn patients (n = 38) aged up to two-and-a-half years old. Dutch reference values are presented in light blue. (**A**) Male pediatric burn patients. (**B**) Female pediatric burn patients. (**C**) Pediatric burn patients with a burn covering less than or equal to ten percent of the total body surface area. (**D**) Pediatric burn patients with a burn covering more than ten percent of the total body surface area.

**Figure 2:**
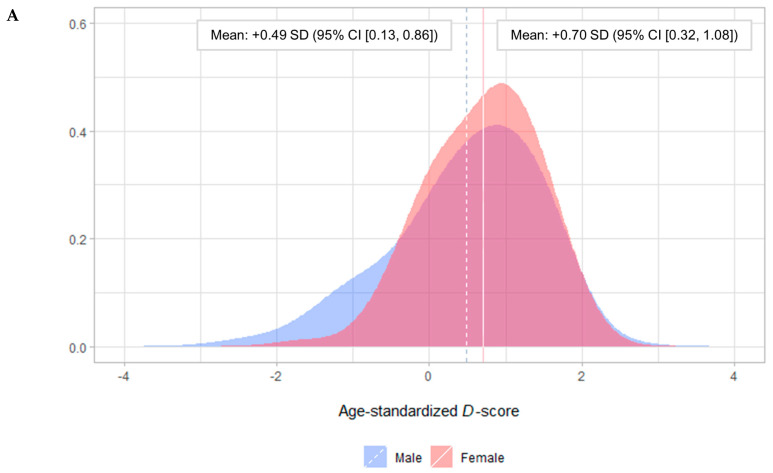

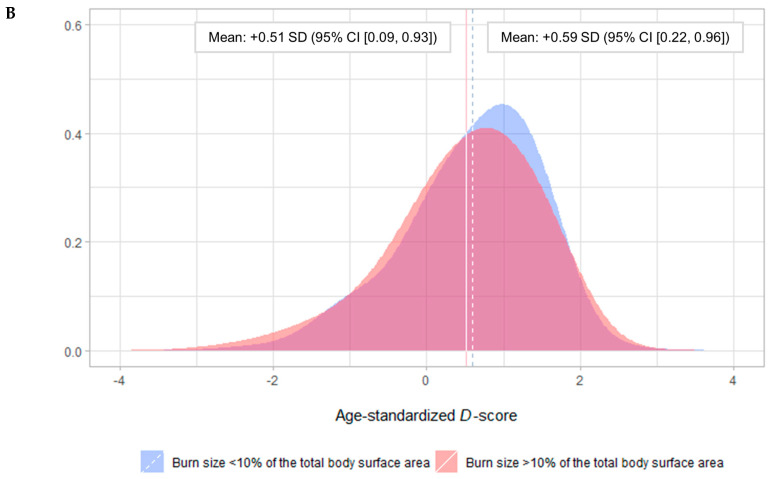
Distribution and mean of the age-standardized *D*-score. (**A**) Stratification by sex. (**B**) Stratification by burn size. SD: standard deviation. 95% CI: 95% confidence interval.

Pediatric burn patients’ individual developmental curves relative to the Dutch reference values are depicted in Figure 1. The mean age-standardized *D*-score was just above the Dutch average: +0.49 SD (95% CI [0.18, 0.80]). The developmental curves of all but one of the pediatric burn patients were on track relative to the Dutch reference values. One patient that was off track concerned a male with a burn covering less than ten percent of the total body surface area and pre-existing developmental delay (Figure 1). The mean of the age-standardized *D*-score did not vary depending on sex (*p* = 0.06; Figure 2A) or burn size (*p* = 0.41; Figure 2B).

## 4. Discussion

Our analyses described the early childhood development of pediatric burn patients using the Dutch Development Instrument, *D*-score, and Dutch reference values. Ninety-five percent (±6.0%) of pediatric burn patients passed each of the age-appropriate developmental milestones at the target age. The mean age-standardized *D*-score was just above the Dutch average (+0.49 SD) and did not vary depending on sex (*p* = 0.06) or burn size (*p* = 0.41). Per domain of the Dutch Development Instrument, pediatric burn patients’ success rate was good to excellent on the age-appropriate developmental milestones related to (i) fine motor skills, adaption, personality, and social behavior; (ii) communication; and (iii) gross motor skills, respectively.

Contrary to our findings, Gorga et al. reported an increase in the number of pediatric burn patients with a suspected developmental delay up to one-year post-burn, in particular within the domain of language [17]. This increase could, however, in part be attributed to the presence of both an increased risk of developmental delay and pre-existing developmental delay within the cohort by Gorga et al. One in two children within the cohort by Gorga et al. was deemed to be at increased risk of developmental delay due to the absence of social and cognitive enrichment in the home environment. Eighty percent of pediatric burn patients within the cohort by Gorga et al. did not attend a school or an organized learning environment. Pediatric burn patients’ parents or guardians had a low rate of educational attainment and often relied on public assistance. Moreover, six percent of pediatric burn patients within the cohort by Gorga et al. had a pre-existing neurological or developmental condition.

Both Nayeb-Hashemi et al. and Kazis et al. reported developmental delay within the domain of motor function [18,22]. Pediatric burn patients’ motor function may be dependent on the extent and severity of the burn. Nayeb-Hashemi et al. included patients aged up to eighteen years old at the time of injury, with a full-thickness burn to the skull. A full-thickness burn to the skull was defined by the presence of necrotic bone extending from the outer table of the skull through the inner table of the skull, with involvement of the attached dura mater. Kazis et al. included pediatric burn patients aged up to five years old, with a burn covering more than twenty percent of the total body surface area, or a burn of any size covering the face, hands, feet, or genitalia. As a result, the cohorts by Nayeb-Hashemi et al. and Kazis et al. included pediatric patients with extensive burns that covered, on average, 39.7% (±26.3) and 18.0% (±18.1) of the total body surface area, respectively, whereas in our study, the median (IQR) burn size and extent of full-thickness burn was 6.0% (4.5–11.9) and 0.0% (0.0–0.0), respectively.

Furthermore, it should be noted that Kazis et al. assessed pediatric patients’ post-burn recovery and development using the BOQ_0–5_ [23]. While the BOQ_0–5_ does include domains that are relevant to early childhood development, such as play, language, fine motor skills, gross motor skills, and behavior, there are significant distinctions that hinder a direct comparison with the Dutch Development Instrument. First, there is the purpose of each instrument: the BOQ_0–5_ is developed to monitor health-related quality of life, and the Dutch Development Instrument is developed to monitor early childhood development [23,35,36]. Second, there is the target audience of each instrument: the BOQ_0–5_ is targeted at pediatric burn patients aged up to five years old, and the Dutch Development Instrument is targeted at children aged up to two-and-a-half years old [23,36]. Third, there is the administration of each instrument: the BOQ_0–5_ is parent-reported, and the Dutch Development instrument is performance-based [23,36].

A major strength of this study included the longitudinal pre- and post-burn data that enabled us to monitor the early childhood development of pediatric burn patients aged up to two-and-a-half years old. An additional strength included the use of the *D*-score and age-standardized *D*-score, which can be calculated from twenty-five common and international instruments; facilitating the comparison of development across different (international) cohorts in the future [27].

However, some limitations need to be addressed. First, the sample consisted of thirty-eight pediatric burn patients. The small sample size increased the risk of selection bias. Yet, according to a non-response analysis in the original cohort, the sample did reflect the pediatric burn population in the Dutch burn centers [24]. Second, Child and Youth Health Care records were not available for 21 out of 59 pediatric burn patients aged up to two-and-a-half years old at the time of injury. Records could either not be located or would have been destroyed if the time limit for storage was reached. Third, the Dutch Development Instrument could be used to monitor the development of children aged up to four years old. However, in practice, it is routinely used to monitor the development of children aged up to two-and-a-half years old. In accordance, Dutch reference values for the *D*-score were limited to children aged up to two-and-a-half years old. Fourth, during data preparation, it was noted that data on age-appropriate developmental milestones were, on occasion, incomplete. Missing data concerned age-appropriate developmental milestones that were not administered due to non-attendance or children’s behavior, including crying and fatigue. In that case, the developmental milestone was likely re-administered at a later age. Consequently, there is the potential risk that pediatric burn patients’ abilities were underestimated. Fifth, unfortunately, the small sample size and reliance on descriptive statistics limited our ability to account for potential effect modification by the location of the burn. Sixth, no data on rehabilitation or psychosocial interventions were available, which could have been informative regarding what is currently working to ensure the age-appropriate development that we are seeing.

In conclusion, among pediatric patients aged up to two-and-a-half years old, with non-full thickness burns, the development was on track relative to the Dutch reference values. Our findings offer valuable first insights into the early childhood development of pediatric burn patients in the Netherlands and may alleviate some parental concerns. Yet, replication and confirmation by future studies is warranted.

## Figures and Tables

**Table 1:** Demographics and clinical characteristics (n = 38).

**Sex, *n* (%)**		
Male	25	(66.0)%
Female	13	(34.0%)
**Age at the time of injury, *median* (IQR)**	1.0	(1.0–2.0)
**Burn size, *median* (IQR)**	6.0	(4.5–11.9)
**Burn size, *n* (%)**		
≤10.0% TBSA	20	(52.6%)
>10.0% TBSA	18	(47.4%)
**Extent of full-thickness burn, *median* (IQR)**	0.0	(0.0–0.0)
**Etiology, *n* (%)**		
Scald	34	(89.5%)
Contact	2	(5.2%)
Fat	2	(5.2%)
**Hospital admission in days, *median* (IQR)**	7.5	(3.0–12.5)
**Mechanical ventilation, *n* (%)**	1	(2.6%)
**Reconstructive surgery, *n* (%)**	1	(2.6%)
**Pre-existing developmental delay, *n* (%)**	1	(2.6%)

IQR: interquartile range. TBSA: total body surface area. Reconstructive surgery: a surgical procedure performed by a plastic surgeon, surgeon, or burn physician after initial wound closure.

## Data Availability

Data are available on request to the corresponding author: Anouk Pijpe (apijpe@rkz.nl).

## Supplementary Materials

The following supporting information can be downloaded at https://www.mdpi.com/article/10.3390/ebj5020012/s1: Supplement S1: Dutch Development Instrument in English.
