# Supplementary material for: The Early Childhood Development of Pediatric Burn Patients
Source: Eur Burn J. 2024 May 14;5(2):145–54. doi: 10.3390/ebj5020012 (PMC11544976; doi:10.3390/ebj5020012)
Supplement: Supplementary file 1 [file ebj-05-00012-s001.zip › ebj-2855026-Supplement S1.pdf]

VAN WIECHEN DEVELOPMENTAL TEST (VWDT) 15-54 months of age

Behavioural state:
0 = Child is awake and alert
1 = Cild seems tired
2 = Kind is fussy
3 = Kind cries continuously
4 = Otherwise: describe under 'remarks'

Social responsiveness::
0 = child is cooperative
1 = child is reserved and needs encouragement
2 = child is shy or reserved without active resistance
3 = child resists and struggles
4 = otherwise: describe under 'remarks'

System of notation:
- Always indicate the calendar age in the appropriate column, also in case of prematurity.
- Use a new column for each test. The column after 11⁄2 year can be used for an extra test.
- Record the result as + or -; if in doubt use -.
- Separately note right and left where asked.
- Observe as much as possible; if needed, information on features marked with (M) (Mentioned) may be gained from parents; if positive, note M.

* Repeat features.

Name:

Dat of birth:

Gestational age: .... weeks

General	15 mth	1½ yr		2 yr	2½ yr	3 yr	3½ yr	4 yr	4½ yr	Remarks
Age										
Behavioural state										
Social responsiveness										
**Fine motor activity, adaptive and personal/social behaviour**	R L	R L	R L	R L	R L	R L	R L	R L	R L	
11. Puts cube in and out of a box										
12. Plays "give and take" (M)										
13. Builds tower of 2 cubes										
14. Explores environment (M)										
15. Builds tower of 3 cubes										
16. Imitates others (M)										
17. Builds tower of 6 cubes										
18. Places round form in form-box										
19. Undresses himself (M)										
20. Imitates building a truck										
21. Places 3 forms in form-box										
22. Imitates drawing vertical line										
23. Imitates building a bridge										
24. Places 4 forms in form-box										
25. Puts on own garment (M)										
26. Copies a circle										
27. Holds pencil with fingers										with (R | L hand)
28. Copies a cross										
**Communication**	15 mth	1½ yr		2 yr	2½ yr	3 yr	3½ yr	4 yr	4½ yr	
37. Says 2 "sound-words" with comprehension (M)										
38. Understands a few daily-used sentences (M)										
39. Says 3 "words" (M)										
40. Understands 'play' orders (M)										
41. Says "sentences" of 2 words (M)										
42. Points at 6 parts of body of a doll (M)										
43. Refers to self using "me" or "I" (M)										
44. Points at 5 pictures in the book										
45. Says "sentences" of 3 or more words (M)										
46. Speech is understood by acquaintances (M)										
47. Talks spontaneously about events at home/playgroup (M)										
48. Asks questions about "who", "what", "where" and "how"(M)										
49. Speech is easily understood by examiner										
50. Asks questions about "how much", "when", "why"? (M)										
51. Understands analogies and oppositions (M)										
**Gross motor activity**	15 mth	1½ yr		2 yr	2½ yr	3 yr	3½ yr	4 yr	4½ yr	
66. Crawls, abdomen off the floor (M)										
67. Walks along (M)										
68.* Walks alone/walks well alone/walks smoothly										(first time:......................mths)
69. Throws ball without falling down										
70. Squats or bends to pick up things										
71. Kicks ball										
72. Can rotate fluently in sitting position										
73. Rides (tri)cycle (M)										
74. Jumps with both feet simultaneously										
75. Can stand on one foot at least 5 seconds										

© 2023-6 Nederlands Centrum Jeugdgezondheid, Utrecht

VAN WIECHEN DEVEOPMENTAL TEST (VWDT) 0-12 (15) months of age.

Behavioural state:
0 = Child is awake and alert
1 = Child seems tired
2 = Child is fussy
3 = Child cries continuously
4 = Otherwise: describe under ‘remarks

System of notation:
• Always indicate the calendar age in the appropriate column, also in case of prematurity.
• Use a new column for each test. The columns after 3 and 6 months can be used for extra tests.
• Record the result as + or -; if in doubt use -.
• Separately note right and left where asked.
• Observe as much as possible; if needed, information on features marked with (M) (Mentioned) may be gained from parents; if positive, note M.
 * Repeat features.

Name:

Date of birth:

Gestational age: .... weeks

**General**	4 wks 1 mth	8wks 2 mth	13 wks 3 mth		26 wks 6 mth		39 wks 9 mth	52 wks 12 mth	65 wks 15 mth	Remarks
Age										
Behavioural state										
	R L	R L	R L	R L	R L	R L	R L	R L	R L	
**Fine motor activity, adaptive and personal/social behaviour**										
1. Eyes fixate										
2. Follows with eyes and head 30°← 0°→ 30 °										
3. Hands occasionally open										
4. Watches own hands (M)										
5. Plays with hands in midline										
6. Supine position: grasps object within reach										
7. Passes cube from hand to hand										
8. Holds cube, grasps another one with other hand										
9. Plays with both feet (M)										
10. Picks up pellet between thumb and index finger										
11. Puts cube in and out of a box										
12. Plays “give and take” (M)										
**Communication**	1 mth.	2 mth	3 mth		6 mth		9 mth	12 mth	15 mth	
29. Reacts when spoken to (M)										
30. Smiles in response (M)										(first time:..........................wks
31. Vocalizes in response (M)										
32. Produces varying sounds (M)										
33. Says “dada-baba” or “gaga”(M)										
34. Babbles while playing (M)										
35. Reacts to verbal request (M)										
36. Makes communicative gestures (M)										
37. Says 2 “sound-words” with comprehension (M)										
38. Understands a few daily-used sentences (M)										
**Gross motor activity**	1 mth	2 mth	3 mth		6 mth		9 mth	12 mth	15 mth	
52. *Moves arms equally frequent										
53.* Moves legs equally frequent										
54. * Stays suspended when lifted under armpits										
55. * Reactions if pulled to sitting										
56. Lifts chin off table for a moment										
57. Lifts head to 45° in prone position										
58. Looks around to side with angle face-table 90°										
59. Flexes or stomps legs while being swung										
60. Rolls over, back and forth (M)										
61. Balances head well while sitting										
62. Sits on buttocks while legs stretched										
63. Sits in stable position, without support										
64. Crawls forward, abdomen on the floor (M)										
65. Pulls up to standing position (M)										
66. Crawls, abdomen off the floor (M)										
67. Walks along (M)										

© 2023-6 Nederlands Centrum Jeugdgezondheid, Utrecht
